# Apoplastic Cell Death-Inducing Proteins of Filamentous Plant Pathogens: Roles in Plant-Pathogen Interactions

**DOI:** 10.3389/fgene.2020.00661

**Published:** 2020-06-26

**Authors:** Ya Li, Yijuan Han, Mengyu Qu, Jia Chen, Xiaofeng Chen, Xueqing Geng, Zonghua Wang, Songbiao Chen

**Affiliations:** ^1^Marine and Agricultural Biotechnology Laboratory, Institute of Oceanography, Minjiang University, Fuzhou, China; ^2^State Key Laboratory of Ecological Pest Control for Fujian and Taiwan Crops, College of Plant Protection, Fujian Agriculture and Forestry University, Fuzhou, China; ^3^School of Agriculture and Biology, Shanghai Jiao Tong University, Shanghai, China

**Keywords:** filamentous phytopathogen, apoplastic effector, cell death-inducing proteins, virulence factor, immune response

## Abstract

Filamentous pathogens, such as phytopathogenic oomycetes and fungi, secrete a remarkable diversity of apoplastic effector proteins to facilitate infection, many of which are able to induce cell death in plants. Over the past decades, over 177 apoplastic cell death-inducing proteins (CDIPs) have been identified in filamentous oomycetes and fungi. An emerging number of studies have demonstrated the role of many apoplastic CDIPs as essential virulence factors. At the same time, apoplastic CDIPs have been documented to be recognized by plant cells as pathogen-associated molecular patterns (PAMPs). The recent findings of extracellular recognition of apoplastic CDIPs by plant leucine-rich repeat-receptor-like proteins (LRR-RLPs) have greatly advanced our understanding of how plants detect them and mount a defense response. This review summarizes the latest advances in identifying apoplastic CDIPs of plant pathogenic oomycetes and fungi, and our current understanding of the dual roles of apoplastic CDIPs in plant-filamentous pathogen interactions.

## Introduction

Filamentous pathogens, such as oomycetes and fungi, are the causal agents of many of the world’s most serious plant diseases, causing extensive annual yield losses of crops worldwide (Giraldo and Valent, 2013; Sánchez-Vallet et al., 2018). Pathogenic oomycetes and fungi secrete a complex repertoire of effector proteins to establish successful interactions with host plants. These oomycete- or fungi-secreted effector proteins may function in the apoplast as well as within plant cells to interfere with host defense by a variety of mechanisms (Dou and Zhou, 2012; Lo Presti and Kahmann, 2017).

Studies from diverse filamentous pathogens have shown that many oomycete or fungal effector proteins possess the ability to induce cell death in plants (Gijzen and Nürnberger, 2006), including avirulence (AVR) proteins which trigger hypersensitive response (HR) upon recognition by cognate resistance (R) proteins (Kamoun, 2006; Ellis et al., 2009), nuclear-localized Crinkling and Necrosis proteins (CRNs) and nucleo-cytoplasmic RxLR proteins with capacity to induce plant cell death (Torto et al., 2003; Amaro et al., 2017), and extracellular cell death-inducing proteins (CDIPs) that function in the plant apoplast (Guo et al., 2019). The role of AVR proteins, CRN and RxLR effectors involved in the interactions of plants with filamentous pathogens have been reviewed extensively (Ellis et al., 2009; Giraldo and Valent, 2013; Amaro et al., 2017; Lo Presti and Kahmann, 2017). Here we focus this review on apoplastic CDIPs of filamentous plant pathogens and their roles in plant-pathogen interactions.

Because cell death plays an important role in the interactions of plants with pathogens, there has been a long-standing interest in the characterization of pathogenic molecules which are able to induce plant cell death (Gijzen and Nürnberger, 2006). Since the characterization of elicitins, a family of conserved small secreted proteins from oomycetes that induce necrosis in *Nicotiana* species, in the 1980s (Billard et al., 1988; Huet and Pernollet, 1989; Ricci et al., 1989), a large number of apoplastic CDIPs have been identified in oomycete and fungal plant pathogens (Tables 1, 2). These apoplastic CDIPs induce plant cell death in a non-race- or non-species-specific manner, and were initially defined or considered as “elicitors” or “toxins” (Gijzen and Nürnberger, 2006; Derevnina et al., 2016). The role of apoplastic CDIPs in the interactions of plants with filamentous pathogens has been controversial for a long time.

**TABLE 1:** Apoplastic cell death-inducing proteins identified in oomycete plant pathogens.

**Species**	**Protein**	**Family**	**Function in virulence/pathogenicity**	**Inducing plant response**	**References**
*P. boehmeriae*^†^	PB90	ND	ND	Induces cell death, JA generation and SA accumulation, and increases of ABA and NO; activates phenylalanine/flavonoid pathways	Wang et al., 2003; Zhang et al., 2004; Chen Q. et al., 2013
*P. cactorum*^†^	Cacto	Elicitin	ND	Induces cell death	Huet et al., 1993
	PcELL1	Elicitin	ND	Induces cell death	Chen X. R. et al., 2014
	PcF	PcF toxin	ND	Induces cell death and *PR* genes expression in tomato	Orsomando et al., 2001
	PcINF1	Elicitin	ND	Induces cell death	Chen et al., 2017
	PcNLP1	NLP	ND	Induces cell death	Chen X. R. et al., 2014
	SCR96, SCR99, SCR121	PcF toxin	SCR96 is important for pathogenicity	Induce cell death	Chen et al., 2016
	SCR113	PcF toxin	ND	Induces cell death	Chen et al., 2017
*P. capsici*^†^	Capsicein	Elicitin	ND	Induces cell death and enhanced defense against *P. nicotianae* in tobacco	Ricci et al., 1989
	Pc11951, Pc107869, Pc109174, Pc118548	NLP	ND	Induce cell death	Chen et al., 2018
	PcINF1	Elicitin	ND	Induces cell death and pepper defense response requiring SGT1/SRC2-1 complex	Liu et al., 2015; Liu Z. Q. et al., 2016
	PcNLP1 to 3, 6 to 10, 13 to 15	NLP	PcNLP2, PcNLP6 and PcNLP14 play important roles in symptom development during *P. capsici* infection	Induce cell death	Feng et al., 2014
	PcPL1, PcPL15, PcPL16, PcPL20	Pectate lyase	The four PcPLs are important virulence factors in *P. capsici*	Induce cell death	Fu et al., 2015
*P. cinnamomi*^†^	Cinnamomin	Elicitin	ND	Induces cell death and protects tobacco against pathogens	Billard et al., 1988; Huet and Pernollet, 1989
*P. colocasiae*^†^	15-kDa glycoprotein	Elicitin	ND	Induces cell death and SAR in taro leaf	Mishra et al., 2009
*P. cryptogea*^†^	Cryptogein	Elicitin	ND	Induces cell death, SAR and defense of tobacco against *P. nicotianae*	Ricci et al., 1989; Galiana et al., 1997; Mikes et al., 1997; Leborgne-Castel et al., 2008; Coursol et al., 2015; Kulik et al., 2015; Ptáčková et al., 2015; Starý et al., 2019
*P. drechsleri*^†^	Dreα, Dreβ	Elicitin	ND	Induces cell death	Huet et al., 1992
*P. hibernalis*^†^	Hibernalin1	Elicitin	ND	Induces cell death	Capasso et al., 2008
*P. infestans*^†^	INF1	Elicitin	Functions as an avirulence factor in the interaction between *N. benthamiana* and *P. infestans*	Triggers HR dependent on HSP70, HSP90 and SGT1; recognized by ELR which associates with BAK1	Huet et al., 1994; Kamoun et al., 1997, 1998; Kanzaki et al., 2003; Huitema et al., 2005; Du et al., 2015
	INF2A, INF2B	Elicitin	ND	INF2A-induced necrosis dependent on SGT1	Huitema et al., 2005
	PiNPP1.1	NLP	ND	Induces HR dependent on SGT1and HSP90; requires COI1, MEK2, NPR1, and TGA2.2 for full cell death inducing activity	Kanneganti et al., 2006
*P. megasperma*^†^	MgMα, MgMβ	Elicitin	ND	Induce cell death	Huet and Pernollet, 1993
	α-megaspermin, β-megaspermin, γ-megaspermin/32 kDa glycoprotein	Elicitin	ND	Induce cell death, *PR* gene expression, and SAR	Baillieul et al., 1995, 2003
*P. palmivora*^†^	Palmivorein	Elicitin	ND	Induces cell death	Churngchow and Rattarasarn, 2000
	High-molecular-weight glycoprotein, broad-molecular-weight glycoprotein, 42-kDa glycoprotein	ND	Promotes infection	Induce cell death and the accumulations of H_2_O_2_, SA, scopoletin, and ABA	Pettongkhao and Churngchow, 2019
*P. parasitica*^†^	CBEL	CBM	ND	Induces cell death; activates defense responses via SA, JA and ET signaling pathways	Mateos et al., 1997; Khatib et al., 2004
*P. parasitica*^†^	PpNLP/NLP_Pp_	NLP	ND	Induces cell death; carries a nlp20 pattern recognized by RLP23 which associates with SOBIR1 and BAK1 complex to trigger immune responses	Fellbrich et al., 2002; Qutob et al., 2006; Ottmann et al., 2009; Böhm et al., 2014; Albert et al., 2015
	OPEL	Thaumatin-like, glycine-rich, and GH16 domains	ND	Induces cell death, expression of SA-responsive genes and PTI marker genes, and plant resistance	Chang et al., 2015
	Parasiticein/parA1/ elicitin 310/elicitin 172	Elicitin	ND	Induces cell death and protection of *C. annuum* and *C. pepo* from *P. capsici*	Nespoulous et al., 1992; Kamoun et al., 1993; Mouton-Perronnet et al., 1995; Capasso et al., 1999
	PPTG_02039, PPTG_14297, PPTG_09966	VmE02 homolog	ND	Induce cell death	Nie J. et al., 2019
*P. sojae*^†^	PsojNIP	NLP	ND	Induces cell death dependent on SGT1 and HSP90	Qutob et al., 2002; Kanneganti et al., 2006
	XEG1	GH12	Acts as an important virulence factor during *P. sojae* infection	Induces cell death; recognized by RXEG1 which associates with SOBIR1 and BAK1 complex to trigger immune responses	Ma et al., 2015, 2017; Wang Y. et al., 2018
*P. syringae*^†^	Syringicin	Elicitin	ND	Induces a hypersensitive response and electrolyte leakage in tobacco	Capasso et al., 2001
*P. aphanidermatum*^†^	PaNie_213_/NLP_Pya_	NLP	ND	Induces cell death and defense responses of carrot cell cultures	Veit et al., 2001; Qutob et al., 2006; Ottmann et al., 2009
S. graminicola*	7-kDa elicitor	ND	ND	Induces cell death and defense responses in cultured cells of *P. glaucum*	Sharathchandra et al., 2006

ND, not determined; NLP, Nep1-like protein; CBM, carbohydrate binding module; GH, glycoside hydrolase; PTI, pattern-triggered immunity; SAR, systemic acquired resistance; *, biotrophic pathogen; ^†^, hemibiotrophic pathogens.

**TABLE 2:** Apoplastic cell death-inducing proteins identified in fungal plant pathogens.

**Species**	**Protein**	**Family**	**Function in virulence/pathogenicity**	**Inducing plant response**	**References**
*A. tenuissima*^‡^	Hrip1	ND	ND	Induces cell death, *PR* gene expression and SAR resistance in tobacco	Kulye et al., 2012
*B. cinerea*^‡^	BC1G_05134	VmE02 homolog	ND	Induces cell death	Nie J. et al., 2019
	BcCFEM1	CFEM	Contributes to virulence	Induces cell death	Zhu et al., 2017b
	BcGs1	GH15	ND	Induces cell death, resistance to multiple types of pathogens	Zhang Y. et al., 2015; Yang et al., 2018a
	BcIEB1	ND	Not required for virulence; protects the fungus against PR5 osmotin	Induces cell death and increased systemic resistance to *B. cinerea* in tobacco	Frías et al., 2016; González et al., 2017
	BcNEP1, BcNEP2	NLP	Not required for virulence	Induce ethylene production, H_2_O_2_ accumulation and cell death	Schouten et al., 2008; Arenas et al., 2010
	BcPG1 to BcPG4, BcPG6	GH28	BcPG1 and BcPG2 were required for virulence	Induce cell death; recognized by RBPG1; RBPG1-mediated response to BcPGs dependent on SOBIR1	ten Have et al., 1998; Kars et al., 2005; Zhang L. et al., 2014
	BcSpl1	CPP	Contributes to virulence	Induces cell death, *PR* gene expression and SAR; requires BAK1 for full cell death inducing activity	Frías et al., 2011, 2013
	BcXYG1	GH12	Contributes to the establishment of infection in early stages	Induces cell death and immune responses dependent on BAK1 and SOBIR1	Zhu et al., 2017a
	BcXyl1	SGNH hydrolase	Contributes to virulence	Triggers PTI responses and resistance to *B. cinerea* and TMV in tobacco and tomato, dependent on BAK1 and SOBIR1	Yang et al., 2018c
	BcXyn11A	GH11	Required for full virulence	Induces cell death and *PR* gene expression	Brito et al., 2006; Noda et al., 2010; Frías et al., 2019
*B. elliptica*^‡^	BeNEP1, BeNEP2	NLP	Not required for virulence	Induce cell death	Staats et al., 2007
*C. fimbriata* f. sp. *platani*^‡^	CP	CPP	ND	Induces cell death; triggers SA- and ET-signaling pathways but not JA-signaling pathway; induces MAPK phosphorylation	Pazzagli et al., 1999; Scala et al., 2004; Baccelli et al., 2014a, b; Luti et al., 2016
*C. populicola*^‡^	Pop1	CPP	ND	Induces cell death, activates defense responses and MAPK phosphorylation	Comparini et al., 2009
*C. falcatum*^†^	CfPDIP1	ND	ND	Elicits cell death, defense in sugarcane and triggers HR in tobacco	Ashwin et al., 2018
	EPL1	CPP	ND	Induces defense and HR in sugarcane and tobacco	Ashwin et al., 2017
*C. gloeosporioides*^†^	CgCP1	CPP	Required for virulence	Induces cell death and accumulation of reactive oxygen species	Wang W. et al., 2018
*C. higginsianum*^†^	ChNIS1	CoNIS1 homolog	ND	Induces cell death	Yoshino et al., 2012
	ChNLP1	NLP	ND	Induces cell death	Kleemann et al., 2012
*C. lindemuthianum*^†^	CLPG1	GH28	ND	Induces cell death	Boudart et al., 2003
*C. orbiculare*^†^	NIS1	CoNIS1	Deletion of NIS1 does not alter virulence	Induces cell death dependent on SGT1 and HSP90, but not RAR1	Yoshino et al., 2012
*D. seriata*^†^	DserNEP1, DserNEP2	NLP	ND	Induce cell death	Cobos et al., 2019
*F. graminearum*^‡^	FgCPP2	CPP	Not essential for virulence	Induces cell death, elicits defense responses, and resistance to *B. cinerea* in *Arabidopsis*	Quarantin et al., 2016, 2019
	FGSG_03624	GH11	Not essential for virulence	Induces cell death and hydrogen peroxide accumulation	Sella et al., 2013
	FGSG_10999, FGSG_11487	FGSG_10999: GH11; FGSG_11487: GH10	ND	Induce cell death	Tundo et al., 2015
*F. oxysporum*^‡^	FocCP1	CPP	Essential for virulence	Triggers HR and SAR in tobacco	Li et al., 2019a; Liu et al., 2019
	Nep1	NLP	Not essential for virulence	Elicits cell death and defense responses	Bailey, 1995; Bailey et al., 2000, 2002, 2005; Bae et al., 2006
	PeFOC1	ND	ND	Induces cell death, and triggers defense response, SAR in tobacco	Li et al., 2019b
*F. virguliforme*^‡^	17-kDa phytotoxin	ND	ND	Induces cell death	Jin et al., 1996
*F. virguliforme*^‡^	FvNIS1	CoNIS1 homolog	Not essential for pathogenicity	Induces cell death	Chang et al., 2016
*G. boninense*^†^	GbNEP	NLP	ND	Induces cell death, production of hydrogen peroxide and superoxide in tobacco dependent on Ca2^+^ activity	Teh et al., 2018
*H. Annosum s.s.*^†^	HaCPL2	CPP	ND	Induces cell death, phytoalexin production and *PR* gene expression	Chen et al., 2015
*M. oryzae*^†^	MoCDI1	RcCDI1 homolog	ND	Induces cell death	Franco-Orozco et al., 2017
	MoCDIP1 to MoCDIP13	MoCDIP1: PbH1; MoCDIP2: CFEM; MoCDIP4: GH61, CBD; MoCDIP8: EEP-1; MoCDIP10: Ferritin-like; MoCDIP11: CFEM	Not essential for virulence	Induce cell death; MoCDIP6 and MoCDIP7 elicit defense responses	Chen S. et al., 2013; Guo et al., 2019
	MoHrip1	AA1 family	Required for virulence	Induces cell death, defense responses by regulating the contents of SA and GA	Chen et al., 2012; Zhang et al., 2017b; Nie H. Z. et al., 2019
	MoHrip2	ND	Required for full virulence	Induces cell death and defense responses	Chen M. et al., 2014; Nie H. et al., 2019
	MoNLP1/Nep1_Mo_, MoNLP2, MoNLP4	NLP	Dispensable for the infection	Induce cell death and defense responses in *N. benthamiana*; MAPK cascade is involved in Nep1_Mo_-triggered plant responses	Zhang et al., 2010, 2012; Teng et al., 2014; Fang et al., 2017
	MoSM1/MSP1	CPP	Contradictory	Induces cell death and defense responses; ectopic expression of MoSM1 in rice and *Arabidopsis* confers enhanced disease resistance	Jeong et al., 2007; Yang et al., 2009; Wang et al., 2016; Hong et al., 2017
*M. perniciosa*^†^	MpCP1	CPP	ND	Induces cell death	Zaparoli et al., 2009
	MpNEP1, MpNEP2	NLP	ND	Induce cell death	Garcia et al., 2007
*N. crassa*^†^	NcCDI1	RcCDI1 homolog	ND	Induces cell death	Franco-Orozco et al., 2017
*P. striiformis* f. sp. tritici*	PstSCR1	(Y/F/W)x(C)	ND	Induces cell death, PTI marker gene expression and enhanced immunity against oomycete pathogens in *N. benthamiana*	Dagvadorj et al., 2017
*P. striiformis* f. sp. tritici*	PSTG_00149	VmE02 homolog	ND	Induces cell death	Nie J. et al., 2019
*R. commune*^†^	RcCDI1	RcCDI1	ND	Induces cell death in *N. benthamiana* dependent on NbBAK1, NbSOBIR1 and NbSGT1	Franco-Orozco et al., 2017
*R. solani*^‡^	AG1IA_05310, AG1IA_07795, AG1IA_09161	AG1IA_05310: CtaG/cox11; AG1IA_07795: inhibitor I9; AG1IA_09161: GT family 2	ND	Induces cell death	Zheng et al., 2013
	RsAG8_06778	Inhibitor I9	ND	Induces cell death	Anderson et al., 2017
	RSAG8_07159	GH10	ND	Induces cell death	Anderson et al., 2017
*S. sclerotiorum*^‡^	sscle_06g048920	VmE02 homolog	ND	Induces cell death	Nie J. et al., 2019
	SsCP1	CPP	Plays an important role in virulence	Induces cell death, SA pathway activation and enhanced resistance	Yang et al., 2018b
	SsCut1	Cutinase	ND	Induces cell death and multiple defense responses in both dicot and monocot species	Zhang H. et al., 2014
U. virens*	UV_44, UV_1423, UV_1533, UV_1338, UV_4040, UV_4753, UV_5436, UV_5517, UV_5851, UV_6205, UV_7115, UV_7823, UV_784	UV_44: peptidase_S8; UV_1423: fungus-specific RNase	ND	Induce cell death	Fang et al., 2016
*V. dahliae*^‡^	PevD1	AA1 family	Required for full virulence	Induces cell death and triggers innate immunity, induces defense response in cotton mediated by an Avr9/Cf-9-INDUCED F-BOX1 (ACIF1)	Wang et al., 2012; Bu et al., 2014; Liu M. et al., 2016; Zhang et al., 2019; Li et al., 2019c
	VD18.5	ND	ND	Induces cell death	Palmer et al., 2005
	VdCP1	CPP	Contributes to virulence	Induces cell death and triggers defense responses	Zhang et al., 2017a
*V. dahliae*^‡^	VdCUT11	Cutinase, CMB1	Contributes to pathogenicity	Induces cell death and triggers defense responses dependent on BAK1 and SOBIR1	Gui et al., 2018
	VdEG1, VdEG3	GH12	Acts as virulence factors	VdEG1 induces cell death and defense responses dependent on BAK1 and SOBIR1; VdEG3 induces cell death and defense responses dependent only on BAK1	Gui et al., 2017
	VdNLP1/VdNEP, VdNLP2	NLP	Dispensable for *V. dahliae* infection in cotton; required for virulence of *V. dahliae* on tomato	Induces cell death and triggers defense responses	Wang et al., 2004; Yao et al., 2011; Zhou et al., 2012; Santhanam et al., 2013
	VdPEL1	Pectate lyase	Contributes to pathogenicity	Induces cell death and triggers defense responses and systemic resistance	Yang et al., 2018d
*V. mali*^‡^	VmE02	ND	Dispensable for virulence	Induces cell death, enhances plant resistance to *S. sclerotiorum* and *P. capsici* dependent on BAK1, SOBIR1, HSP90 and SGT1	Nie J. et al., 2019
*Z. tritici*^‡^	MgNLP	NLP	Dispensable for virulence	Induces cell death and immune responses dependent on BAK1 and SOBIR1	Motteram et al., 2009; Kettles et al., 2017
	Zt1, Zt2, Zt4, Zt5, Zt7 to Zt15	ND	ND	Induce cell death in **N. benthamiana** dependent on NbBAK1 and NbSOBIR1	Kettles et al., 2017
	ZtCDI1	RcCDI1 homolog	ND	Induces cell death	Franco-Orozco et al., 2017
	ZtNIP1, ZtNIP2	NLP	ND	Induce cell death	M’Barek et al., 2015

ND, not determined; NLP, Nep1-like protein; CBM, carbohydrate binding module; GH, glycoside hydrolase; CFEM, common in fungal extracellular membrane; CPP, cerato-platanin protein; AA1, Alt a 1; HR, hypersensitive response; PTI, pattern-triggered immunity; SAR, systemic acquired resistance; *, biotrophic pathogens; ^†^, hemibiotrophic pathogens; ^‡^, necrotrophic pathogens.

Over the past decades, tremendous progress has been made in understanding the biological functions of apoplastic CDIPs in filamentous oomycetes and fungi, such as their contribution to pathogen virulence and being recognized by plant cells. These findings have greatly enriched our knowledge on the roles of apoplastic CDIPs as virulence factors and how plants detect them and mount a defense response. In this review, we summarize the latest advances in identifying apoplastic CDIP effectors in plant pathogenic oomycetes and fungi, and our current understanding of the dual roles of apoplastic CDIPs in plant-filamentous pathogen interactions.

## Apoplastic CDIPs Identified in Plant Pathogenic Oomycetes and Fungi

### Apoplastic CDIPs Identified in Phytopathogenic Oomycetes

The purification and identification of extracellular proteins from phytopathogenic oomycetes that induce plant cell death began in the 1980s. Pernollet and colleagues purified three extracellular proteins, namely capsicein, cinnamomin and cryptogein, in culture filtrates of *Phytophthora capsici*, *P. cinnamomi* and *P. cryptogea*, respectively (Billard et al., 1988; Huet and Pernollet, 1989; Ricci et al., 1989). Determination of the amino acid sequences of these proteins led to definition of a novel protein family, called elicitins (Derevnina et al., 2016). Over the past decades, building on advances in molecular biology and genome sequencing, significant progress has been made in identifying extracellular proteins from phytopathogenic oomycetes that induce cell death in plants. To date, over 62 apoplastic CDIPs have been identified in 16 oomycete species (Table 1). While a few identified oomycete apoplastic CDIPs belong to PcF toxin, pectate lyase and glycoside hydrolase (GH) families or with no conserved domains, the majority are elicitins or Nep1 (necrosis- and ethylene-inducing peptide 1)-like proteins (NLPs) (Tables 1, 3).

**TABLE 3:** Major protein families of apoplastic cell death-inducing proteins identified in oomycete and fungal plant pathogens.

**Protein family**	**Predicted functional category**	**Taxon**		**Representative CDIPs**	**Plant cell surface receptor**	**Co-receptor and downstream component in plant cell**	**Representative references**
		**Oomycete**	**Fungi**				
CE	Cutinase	+	+	SsCut1, VdCUT11	Unknown	BAK1, SOBIR1	Zhang H. et al., 2014; Gui et al., 2018
CFEM	ND	−	+	BcCFEM1, MoCDIP2, MoCDIP11	Unknown	Unknown	Kulkarni et al., 2003; Chen S. et al., 2013; Zhu et al., 2017b
CPP	ND	−	+	BcSpl1, CP, MoSM1/MSP1	Unknown	BAK1	Pazzagli et al., 1999; Jeong et al., 2007; Frías et al., 2011
Elicitin	ND	+	−	Cryptogein, INF1	ELR	BAK1, HSP70, HSP90, NbLRK1, SGT1, SRC2-1	Ricci et al., 1989; Huet et al., 1994; Kamoun et al., 1997; Kanzaki et al., 2008; Du et al., 2015; Liu et al., 2015
GH10	Xylanase	ND	+	RSAG8_07159; FGSG_11487	Unknown	Unknown	Anderson et al., 2017
GH11	Xylanase	ND	+	BcXyn11A, EIX*, FGSG_03624, FGSG_10999	LeEix1, LeEix2	BAK1	Fuchs et al., 1989; Ron and Avni, 2004; Brito et al., 2006; Bar et al., 2010; Sella et al., 2013
GH12	Xylanase	+	+	XEG1, BcXYG1, VdEG1, VdEG3	RXEG1	BAK1, SOBIR1	Ma et al., 2015; Gui et al., 2017; Zhu et al., 2017a; Wang Y. et al., 2018
GH15	Glucan 1,4-alpha-glucosidase	ND	+	BcGs1	Unknown	Unknown	Zhang Y. et al., 2015; Yang et al., 2018a
GH16	ND	+	−	OPEL	Unknown	Unknown	Chang et al., 2015
GH28	Polygalacturonase	ND	+	BcPG1 to 4, BcPG6, CLPG1	RBPG1	SOBIR1	ten Have et al., 1998; Boudart et al., 2003; Kars et al., 2005; Zhang L. et al., 2014
GH61	ND	ND	+	MoCDIP4	Unknown	Unknown	Chen Q. et al., 2013
NLP	ND	+	+	PpNLP/NLP_Pp_, PaNie_213_/NLP_Pya_	RLP23	BAK1, COI1, HSP90, MEK2, NPR1, SGT1, SOBIR1 and TGA2.2	Bailey, 1995; Fellbrich et al., 2002; Qutob et al., 2006; Ottmann et al., 2009; Böhm et al., 2014; Albert et al., 2015
PL	Pectate lyase	+	+	PcPL1, PcPL15, PcPL16, PcPL20, VdPEL1	Unknown	Unknown	Fu et al., 2015; Yang et al., 2018d

ND, not determined; +, present; −, absent; CE, carbohydrate esterase; CFEM, common in fungal extracellular membrane; CPP, cerato-platanin protein; GH, glycoside hydrolase; NLP, Nep1-like protein; PL, polysaccharide lyase. *, EIX (ethylene-inducing xylanase) is an apoplastic CDIP identified from a non-pathogenic fungi *Trichoderma viride*.

### Apoplastic CDIPs Identified in Phytopathogenic Fungi

Similarly, the availability of genome sequences of fungi has led to a rapid identification of apoplastic CIPDs from phytopathogenic fungi. To date, over 115 apoplastic CDIPs have been identified in 27 fungal species, including biotrophic, hemibiotrophic and necrotrophic pathogens (Table 2). The identified fungi apoplastic CDIPs include CFEM (common in fungal extracellular membrane)-containing proteins, cerato-platanin proteins (CPPs), GHs, NLPs, cutinases, and pectate lyases, and some proteins with other domains or with no conserved domains (Tables 2, 3). Among the identified oomycete and fungus apoplastic CDIPs, GHs, NLPs, Pectate lyases, and VmE02 homologues are widely distributed across oomycetes and fungi (Tables 1–3). On the other hand, many CDIPs are oomycete-specific or fungi-specific. For example, elicitins and elicitin-like proteins are unique to oomycetes, whereas CFEM-containing proteins and CPPs are unique to fungi (Table 3).

## Major Protein Families of Oomycete and Fungus Apoplastic CDIPs

### CFEM Containing CDIPs

The CFEM domain, which contains eight conserved cysteines, is unique to fungi (Kulkarni et al., 2003; Zhang Z. N. et al., 2015). CFEM was first identified in a *M. oryzae* MAC1-interacting protein, ACI1 (Kulkarni et al., 2003). CFEM containing proteins are widely distributed in fungi (Zhang Z. N. et al., 2015). While some of CFEM containing proteins have been identified to have prominent roles in pathogenicity or virulence, such as MoPth11 and its ortholog FGRRES_16221 in *Magnaporthe oryzae* and *Fusarium graminearum*, respectively (Kou et al., 2017; Dilks et al., 2019), and CgCcw14, CgMam3 in *Candida glabrata* (Srivastava et al., 2014), several CFEM containing proteins have been identified to possess cell death-inducing activity.

The CFEM-containing protein BcCFEM1 from *Botrytis cinerea*, contains a CFEM domain at the N-terminus, and a glycosylphosphatidylinositol (GPI) anchored site at the C-terminus. *BcCFEM1* was induced and expressed at high levels during early stages of infection on bean leaves. Targeted disruption of the *BcCFEM1* gene reduced virulence, conidiation and stress tolerance in *B. cinerea*. Transient expression of BcCFEM1 in *Nicotiana benthamiana* leaves triggered obvious chlorosis (Zhu et al., 2017b), suggesting the involvement of BcCFEM1 in eliciting plant responses.

The *M. oryzae* genome harbors 19 proteins with the CFEM domain (Kou et al., 2017). Two CFEM-containing proteins MoCDIP2 and MoCDIP11 have been identified to induce cell in the non-host *N. benthamiana* and host rice cells (Chen S. et al., 2013; Guo et al., 2019). MoCDIP2 contains a CFEM domain at the N-terminus, and a GPI-anchored site at the C-terminus, whereas MoCDIP11 contains only a CFEM domain at the N-terminus. Transient expression assays in rice protoplasts or *N. benthamiana* leaves revealed that the signal peptides that led the secretion of proteins, were required for cell death inducing activity of MoCDIP2 and MoCDIP11, indicating that both two effectors function in the apoplast (Chen S. et al., 2013; Guo et al., 2019).

### CPP Family CIDPs

CPPs are small secreted cysteine-rich proteins that widely occur in filamentous fungi but not in bacteria, oomycetes, plants, or animals (Chen H. et al., 2013). CPPs have many aspects in common with expansins. Structural analysis revealed that CPPs have a double ψβ-barrel similar to the D1 domain of expansins (de Oliveira et al., 2011). Moreover, similar to expansins, CPPs possess properties to weaken cellulose (de O Barsottini et al., 2013; Baccelli et al., 2014b). Many CPPs have been shown to function as virulence factors in fungi, and on the other hand are able to induce cell death and elicit defense response in plants (Pazzagli et al., 2014).

The first CPP family CIDP, CP, was identified from the culture filtrates of *Ceratocystis fimbriata* f. sp. *platani*, the causal agent of the plane canker stain (Pazzagli et al., 1999). CP induced cell death in host and non-host plants, activated phytoalexin synthesis, pathogenesis-related (*PR*) gene expression, and mitogen-activated protein kinase (MAPK) phosphorylation, and triggered salicylic acid (SA) and ethylene (ET)-signaling pathways (Pazzagli et al., 1999; Scala et al., 2004; Baccelli et al., 2014a; Luti et al., 2016).

Till now, in addition to CP, 11 more CPP family CIDPs have been identified from different fungal pathogens (Table 2), such as BcSpl1 in *B. cinerea* (Frías et al., 2011, 2013), EPL1 in *Colletotrichum falcatum* (Ashwin et al., 2017), CgCP1 in *Colletotrichum gloeosporioides* (Wang W. et al., 2018), Pop1 in *Ceratocystis populicola* (Comparini et al., 2009), FgCPP2 in *F. graminearum* (Quarantin et al., 2016, 2019), FocCP1 in *Fusarium oxysporum* (Li et al., 2019a; Liu et al., 2019), HaCPL2 in *Heterobasidion annosum sensu stricto* (Chen et al., 2015), MoSM1/MSP1 (Jeong et al., 2007; Yang et al., 2009; Wang et al., 2016; Hong et al., 2017) in *M. oryzae*, MpCP1 in *Moniliophthora perniciosa* (Zaparoli et al., 2009), SsCP1 in *Sclerotinia sclerotiorum* (Yang et al., 2018b), and VdCP1 in *Verticillium dahliae* (Zhang et al., 2017a). Similar to CP, these 11 CPP family CIDPs induced strong necrosis and elicited defense responses in plants. For example, BcSpl1 induced strong necrosis, electrolyte leakage, cytoplasm shrinkage, and *PR* gene expression in plants including tomato, tobacco and *Arabidopsis*. In addition, BcSpl1 elicited systemic acquired resistance (SAR) in tobacco against *Pseudomonas syringae and B. cinerea* (Frías et al., 2011). The CPP family CIDPs possess similar biological properties as that of CP. For example, Pop1 and VdCP1 possessed chitin-binding properties (Baccelli et al., 2014b; Zhang et al., 2017a), and Pop1 and FgCPP2 exhibited the ability of loosening cellulose substrates and enhancing fungal cellulase activity in an expansin-like manner as well as CP (Baccelli et al., 2014b; Quarantin et al., 2016, 2019).

### Elicitin and Elicitin-Like CDIPs

Elicitins are a family of small, highly conserved proteins secreted by *Phytophthora* and *Pythium* species (Derevnina et al., 2016). The first three elicitins, cryptogein, cinnamomin and capsicein were identified from the culture filtrates of *P. cryptogea*, *P. cinnamomi* and *P. capsici*, respectively (Billard et al., 1988; Huet and Pernollet, 1989; Ricci et al., 1989). Sequence analysis revealed that these elicitins share a conserved domain of 98 amino acids, which contains six cysteine residues at conserved positions forming three disulphide bridges (Boissy et al., 1996). Further studies revealed that elicitins are encoded by complex gene families (Kamoun et al., 1993; Panabieres et al., 1995; Jiang et al., 2006). In addition, *Phytophthora* and *Pythium* genomes contain a number of elicitin-like proteins possessing diverse, shorter or longer elicitin domains (Jiang et al., 2006).

Over 22 elicitin and elicitin-like CDIPs that induce cell death in certain plant species such as *Nicotiana* species and some Brassicaceae cultivars have been identified from different *Phytophthora* pathogens (Table 1). Among the elicitin and elicitin-like CDIPs, *β*-cryptogein and INF1 have been widely studied for their roles in the interactions of *Phytophthora* pathogens with plants. Cryptogein induced necrosis on tobacco leaves, triggered SAR and enhanced disease resistance in tobacco. Upon the SAR induction, the expression of the plant extracellular S-like RNase *NE* gene and its RNase activity were highly up-regulated, indicating that NE possibly associated with the cryptogein-induced SAR (Galiana et al., 1997). Cryptogein promoted the movement of plant respiratory burst oxidase homologues (RBOHs) from the Golgi cisternae to the plasma membrane, which may play a fundamental role for ROS production (Noirot et al., 2014). Cryptogein induced production of ROS, which was differentially regulated by the sphingolipid long-chain bases (LCBs) and their phosphorylated derivatives (LCB-Ps) in tobacco cells (Coursol et al., 2015). In addition, cryptogein induced production of NO, partly dependent on the ROS-dependent pathway, indicating that the defense responses induced by cryptogein involving interaction of the NO and ROS signaling pathways (Kulik et al., 2015). INF1 induced cell death on tobacco and potato leaves with the necrotic activity higher than that of other *a*-elicitins, such as cacto, parasiticein, capsicein and cryptogein, but less than that of *β*-cryptogein (Huet et al., 1994). INF1 induced the expression of *chitinase*, *β-1,3-glucanase*, *phenylalanine ammonia-lyase* (*PAL*), and *PR1* genes, and rapid accumulation of H_2_O_2_ in tobacco (Sasabe et al., 2000; Huitema et al., 2005). INF1 induced the expression of *NbrbohB* in *N. benthamiana*, and silencing of *NbrbohA* or *NbrbohB* led to a reduction and delay of the necrotic reaction triggered by INF1 (Yoshioka et al., 2003), suggesting that INF1-induced cell death was dependent on the ROS burst.

Elicitins are able to bind sterols, phospholipids or fatty acids, and transport them between biological membranes (Mikes et al., 1997). However, the lipid-binding ability did not influence elicitin-induced response in tobacco. Investigations based on the mutant proteins of cryptogein with limited abilities to bind sterols revealed that induction of ROS synthesis, cytosol acidification and cell death in tobacco cells were not correlated with the sterol-binding abilities of the cryptogein proteins (Dokládal et al., 2012; Ptáčková et al., 2015).

Elicitins do not induce cell death in tomato plants. However, elicitins could induce immune responses in tomato, and enhance plant resistance against *Phytophthora* spp., bacterial wilt disease, and powdery mildew (Picard et al., 2000; Kawamura et al., 2009; Starý et al., 2019). INF1 induced the expression of jasmonic acid (JA)-responsive *PR-6*, *LeATL6* and *LOX-E*, and ET-responsive *PR-2b* and *ERF2*, but not SA-responsive *PR-1a* and *PR-2a* in tomato leaves (Kawamura et al., 2009). Consistently, Starý et al. (2019) showed that cryptogein induced defense responses without cell death in tomato through JA- and ET-signaling pathways, but not SA-signaling pathway. These results indicated that elicitins triggered different signaling pathways between tobacco and tomato.

### NLP Family CDIPs

NLP family proteins are characterized by the presence of a common NPP1 (necrosis-inducing *Phytophthora* protein) domain. This family is widely distributed across taxa including oomycetes, fungi, and bacteria (Gijzen and Nürnberger, 2006). The first NLP protein, namely Nep1, was identified from culture filtrates of *F. oxysporum* (Bailey, 1995), and has been shown to induce necrosis in plants, such as *Erythroxylum coca*, *Theobroma cacao* and *Arabidopsis*, with activating *PR* gene expression, ROS production and other general defense response (Bailey et al., 2005; Bae et al., 2006). To date, over 39 NLP family CDIPs have been identified in phytopathogenic oomycetes and fungi. Among them, 20 NLP family CDIPs were from phytopathogenic oomycetes (Table 1), and the remaining 19 NLP CDIPs were from phytopathogenic fungi (Table 2).

NLP family CDIPs have been confirmed to induce cell death in dicot but not monocot plants. Among the identified NLP family CDIPs, PpNLP/NLP_Pp_, PsojNIP and PaNie_213_/NLP_Pya_ have been extensively studied in the context of inducing cell death and immune responses in dicot plants. PpNLP/NLP_Pp_ induced necrosis in *Arabidopsis*, and activated *PR* genes expression, ROS production, callose apposition (Fellbrich et al., 2002), as well as the posttranslational expression of mitogen-activated protein kinase and production of nitric oxide and phytoalexin camalexin, suggesting dual roles of PpNLP/NLP_Pp_ as both toxin-like virulence factors and plant innate immunity triggers (Qutob et al., 2006). Determining and modeling of the structures of PaNie_213_/NLP_Pya_, PpNLP/NLP_Pp_, and a PccNLP protein from the phytopathogenic bacterium *Pectobacterium carotovorum*, revealed that NLPs displayed identical toxin folds which contributed to host infection and plant defense gene activation, suggesting that a common fold of the cytolytic toxin is required for both pathogen virulence and plant immunity activation (Ottmann et al., 2009). Böhm et al. (2014) further identified and characterized a pattern of 20 amino acid residues (nlp20) of cytotoxic NLPs that triggered immunity associated plant defenses in certain dicot plants.

Based on the finding that NLPs display a striking similarity to cytolytic sphingomyelin-binding actinoporins (Ottmann et al., 2009), glycosylinositol phosphorylceramide (GIPC) sphingolipids in eudicot plants were further shown to be bound by NLPs. The inositol phosphorylceramide in GIPCs was covalently bound to glucuronic acid and variable terminal hexoses which were different between eudicots and monocots. Eudicot GPICs typically carried two terminal sugars (series A), while monocots GIPCs bore three terminal sugars (series B) (Cacas et al., 2013). The absence of series A GIPCs lead to insensitivity to NLPs of monocot plants. Consistently, *Arabidopsis* mutants with altered GIPC composition suffered less cell death than the wild type upon NLP infiltration (Lenarčič et al., 2017). These results thus explained host specificity of cell death induced by NLPs in eudicot plants (Van den Ackerveken, 2017).

### Cell Wall-Degrading Enzyme Family CDIPs

Phytopathogenic oomycetes and fungi secrete a large amount of effector proteins related to plant cell wall degradation, such as enzymes to degrade cellulose, xylan, pectin, etc. (Kubicek et al., 2014). Certain cell wall-degrading enzymes (CWDEs) have been proved to be associated with pathogen virulence, and some induced cell death and trigged defense responses in plants. CWDE family CDIPs have been identified in carbohydrate esterase (CE5), glycoside hydrolase (GH) and polysaccharide lyase (PL) families (Tables 1–3).

With functions to degrade plant cuticle or suberin polymers, the CE family cutinases has been associated with important roles in filamentous pathogen-plant interactions. Two cutinase family CDIPs, SsCut1 and VdCUT11 have been identified in *S. sclerotiorum* and *V. dahliae* (Zhang H. et al., 2014; Gui et al., 2018), respectively. SsCut1 induced cell death in both dicot and monocot species and activated plant resistance against *S. sclerotiorum*, *P. nicotianae* and *Phytophthora soja*e (Zhang H. et al., 2014). Further, SsCut1-induced cell death along with stomatal closure, ROS burst and NO production, was suppressed by silencing of a C_2_H_2_-type zinc finger gene *NbCZF1* in *N. benthamiana*, showing that SsCut1-triggered defense could be mediated by NbCZF1-ROS-NO pathway (Zhang et al., 2016). VdCUT11 induced cell death and defense responses in *N. benthamiana*, cotton, and tomato plants, and the enzymatic activity was required for its cell death-inducing activity (Gui et al., 2018).

GHs hydrolyze the glycosidic bond between carbohydrates or between a carbohydrate and a noncarbohydrate moiety through acid catalysis (Zhao et al., 2013). About 18 GH domain-containing CDIPs have been identified (Table 3). The GH family CDIPs induced cell death as well as defense responses in host and nonhost plants. For example, BcXyn11A induced ROS burst, electrolyte leakage, cytoplasm shrinkage and *PR* gene expression, and these effects were dependent on its short 25-residue peptide (Frías et al., 2019). BcXYG1 triggered pattern-triggered immunity (PTI) response and systemic resistance in bean (Zhu et al., 2017a). PsXEG1 induced disease resistance in *N. benthamiana* and soybean (Ma et al., 2015). BcGs1 induced systemic resistance in tobacco and tomato against *B. cinerea*, *Phytophthora syringae* and tobacco mosaic virus (Zhang Y. et al., 2015). Furthermore, BcGs1 triggered ROS burst, PAL and peroxidase (POD) enzyme activity, and lignin accumulation in tomato (Yang et al., 2018a). Many of the GH family CDIPs have been shown to possess hydrolase activity. While hydrolase activity of BcPG2 was required for its cell death induction (Kars et al., 2005), the enzymatic activity of most identified GH family CDIPs was not necessary for cell death inducing activity. For example, the cell death inducing activity of Xyn11A, PsXEG1, BcXYG1, VdEG1 and VdEG3 was independent of their enzymatic activity (Brito et al., 2006; Ma et al., 2015; Gui et al., 2017; Zhu et al., 2017a).

The PL family pectate lyases play a critical role in pectin degradation. Four pectate lyases PcPL1, PcPL15, PcPL16 and PcPL20 in *P. capsici*, and one pectate lyase VdPEL1 in *V. dahliae* have been identified to have cell death inducing activity in plants (Fu et al., 2015; Yang et al., 2018d). *PcPL1*, *PcPL15*, *PcPL16* and *PcPL20* were highly expressed in *P. capsici* during infection of pepper, and transient expression of the four PcPLs induced severe cell death in pepper leaves (Fu et al., 2015). VdPEL1 induced cell death in *N. benthamiana*, tomato, soybean and cotton, and triggered defense responses and systemic resistance to *B. cinerea* and *V. dahliae* in *N. benthamiana* and cotton plants. Furthermore, the enzymatic activity was found to be necessary for cell death-inducing activity (Yang et al., 2018d).

Besides, a SGNH hydrolase subfamily protein BcXyl1 was identified to induce cell death in *N. benthamiana*, tomato, soybean and cotton (Yang et al., 2018c). BcXyl1 exhibited xylanase activity, but its cell death inducing activity was independent of the enzymatic activity. BcXyl1 triggered plant PTI responses with a pattern of 26-amino acid peptide.

### CDIP Families With Other Conserved Domains or Without Conserved Domain

Besides the above-mentioned CDIP families, a number of apoplastic CDIPs contain other conserved domains or no conserved domain (Tables 1, 2), indicating the incredible diversity of apoplastic CDIPs secreted by oomycetes and fungi. On the other hand, many of these apoplastic CDIPs are widely distributed across microbial taxa or different pathogen species and the homologs can induce cell death and defense responses in different plant species, indicating that recognition of these proteins is evolutionarily conserved. For example, the *Valsa mali* small cysteine-rich protein VmE02 induces cell death in *N. benthamiana*, tomato, pepper, *Arabidopsis* and apple and enhances resistance in *N. benthamiana* against *S. sclerotiorum* and *P. capsic* (Nie J. et al., 2019). VmE02 is widely conserved across oomycete and fungal species, and the homologs can induce cell death in *N. benthamiana*. Similarly, the *Colletotrichum orbiculare* NIS1 and its homologs from *Colletotrichum higginsianum* and *Fusarium virguliforme* (Yoshino et al., 2012; Chang et al., 2016), and the *Rhynchosporium commune* RcCDI1 and its homologues from *M. oryzae*, *Neurospora crassa* and *Zymoseptoria tritici* (Franco-Orozco et al., 2017), induced cell death in *N. benthamiana*. These results clearly supported that, although the physiological properties remain unknown, these apoplastic CDIPs are recognized as conserved patterns that induce defense responses in plants.

## Apoplastic CDIPs Contribute to Pathogen Virulence

Phytopathogenic oomycetes and fungi initially colonize in the plant apoplast or extracellular space, and subsequently penetrate host cells. Therefore, the apoplastic effectors secreted by oomycetes and fungi are likely the primary weapons of filamentous plant pathogens. Despite the diversity of lifestyles as biotrophic, hemibiotrophic or necrotrophic, filamentous oomycetes and fungi secrete a high number of apoplastic CDIPs. Biotrophic pathogens feed on living plant cells (Giraldo and Valent, 2013). Whether apoplastic CDIPs of biotrophic pathogens actually cause plant cell death or function as important virulence factors during a natural infection remain to be determined (Tables 1, 2). In contrast, necrotrophic pathogens thrive on dead host tissues and take advantage of CDIP-triggered plant cell death. For example, NLP-triggered necrosis could aid infection by necrotrophic pathogens (Van den Ackerveken, 2017). Hemibiotrophic pathogens combine a biotrophic phase in early stages with a necrotrophic phage during later infection stages. Many apoplastic CDIPs of hemibiotrophic pathogens have been found to be highly expressed at late infection stages, suggesting that they contribute to the necrotrophic growth of hemibiotrophic pathogens (Qutob et al., 2002; Kelley et al., 2010).

Functional analysis based on overexpression, deletion or silencing of genes encoding apoplastic CDIPs have functionally proved many of them as important virulence factors in hemibiotrophic and necrotrophic pathogens (Tables 1, 2). Among the 62 identified oomycete apoplastic CDIPs, 12 have been proven to function as virulence factors that are required for pathogenicity or contribute to virulence. The *P. cactorum* SCR96 belongs to the PcF toxin family. Silencing of the *scr96* gene in *Phytophthora cactorum* caused loss of pathogenicity on host plants, indicating that SCR96 is required for pathogenicity (Chen et al., 2016). In *P. capsici*, silencing of the genes encoding NLP-like proteins PcNLP2, PcNLP6 or PcNLP14, and the genes encoding pectate lyases PcPL1, PcPL15, PcPL16 or PcPL20 caused significantly reduced virulence on pepper (Feng et al., 2014; Fu et al., 2015), demonstrating the important roles of these apoplastic CDIPs in pathogen virulence. In *Phytophthora palmivora*, three fractions isolated from culture filtrates including high-molecular-weight glycoprotein, broad-molecular-weight glycoprotein and 42-kDa glycoprotein were observed to promote *P. palmivora* infection of rubber tree leaves (Pettongkhao and Churngchow, 2019). However, the virulence role of these three glycoproteins remains to be genetically confirmed. The *P. sojae* XEG1, a GH12 family CDIP, functions as a major virulence factor during infection. Both silencing and overexpression of the *PsXEG1* gene in *P. sojae* severely impaired virulence (Ma et al., 2015). Interestingly, *P. sojae* also secretes a PsXLP1 (PsXEG1-like) apoplastic effector with a truncated GH12 domain functioning as a decoy to shield XEG1-mediated virulence (Ma et al., 2017).

Among the 115 identified fungi apoplastic CDIPs, 16 have been proven to be required for pathogenicity or contribute to virulence of both hemibiotrophic and necrotrophic pathogens. In *B. cinerea*, targeted deletion of the genes encoding BcCFEM1 (Zhu et al., 2017b), BcSpl1 (Frías et al., 2011), BcXyl1 (Yang et al., 2018c), or BcXyn11A (Brito et al., 2006) caused severely compromised virulence, suggesting that these CDIPs were important virulence factors of *B. cinerea*. Overexpression and deletion of *BcXYG1* did not significantly affect *B. cinerea* infection on bean leaves. However, the *BcXYG1* overexpression strains produced significantly earlier and more intense local necrosis, suggesting that BcXYG1 contributes to the establishment of infection in early stages (Zhu et al., 2017a). The *C. gloeosporioides* CgCP1 belongs to the CPP family. Knock-out of *CgCP1* in *C. gloeosporioides* significantly reduced infection on rubber tree leaves (Wang W. et al., 2018). Similar to BcSpl1 and CgCP1, the CPP family CIDPs FocCP1, SsCP1 and VdCP1 function as important virulence factors. Deletion of *FocCP1* in *F. oxysporum* (Liu et al., 2019), *SsCP1* in *S. sclerotiorum* (Yang et al., 2018b), or *VdCP1* in *V. dahliae* (Zhang et al., 2017a) caused significantly reduced virulence of pathogens on hosts banana, *Arabidopsis* or cotton, respectively. In *M. oryzae*, deletion of *mohrip1*, or *mohrip2* remarkably compromised fungal virulence on rice (Nie H. et al., 2019; Nie H. Z. et al., 2019). MoHrip1 belongs to the Alt a 1 (AA1) family (Zhang et al., 2017b). Similarly, the *V. dahliae* PevD1, an AA1 family CDIP, is required for full virulence of *V. dahliae* on hosts (Zhang et al., 2019). In addition to VdCP1 and PevD1, VdCUT11, VdEG1, VdEG3, VdNLP1, VdNLP2, and VdPEL1 have been shown to play important roles in virulence of *V. dahliae*. Targeted deletion of *VdCUT11* (Gui et al., 2018), *VdEG1*, *VdEG3* (Gui et al., 2017), or *VdPEL1* (Yang et al., 2018d) significantly compromised virulence of *V. dahliae* on cotton plants. Interestingly, while both VdNLP1 and VdNLP2 appear to be dispensable for *V. dahliae* infection in cotton plants (Zhou et al., 2012), *VdNLP1* as well as *VdNLP2* deletion strains were found to be significantly less pathogenic on tomato and Arabidopsis (Santhanam et al., 2013), demonstrating the functional diversification of the two NLP family CDIPs in virulence of *V. dahliae*.

Pathogenicity assays also revealed that many apoplastic CDIPs were not required for fungal pathogenicity or virulence. Targeted deletion of the genes encoding these proteins did not impair the virulence of pathogens (Table 2). One possibility why these apoplastic CDIPs were dispensable for virulence could be due to the functional redundancy with other apoplastic effectors (Yoshino et al., 2012; Guo et al., 2019).

## Recognition of Apoplastic CDIPs in Plants: Cell Surface Receptors and Downstream Components

In recent years, there have been breakthroughs in the identification of cell surface receptors recognizing apoplastic CDIPs. By screening T-DNA insertion mutants and natural accessions of *Arabidopsis*, Albert et al. (2015) identified T-DNA insertion alleles of *RLP23* and *Arabidopsis* accessions carrying a frameshift mutation of *RLP23* that were insensitive to the conserved nlp20 pattern found in most NLPs. *RLP23* encodes a leucine-rich repeat-receptor-like protein (LRR-RLP), which binds extracellularly to nlp20, thereby mediating NLP-elicited immune response in *Arabidopsis* (Albert et al., 2015). Transgenic potato plants expressing RLP23 displayed enhanced resistance to oomycete and fungal pathogens, such as *P. infestans* and *S. sclerotiorum* (Albert et al., 2015), further supporting that RLP23 confers protection to oomycete and fungal pathogens. More recently, RXEG1 (Response to XEG1), an LRR-RLP that specifically recognizes the GH12 family CDIP XEG1, has been identified from *N. benthamiana* through a high-throughput virus-induced gene silencing (VIGS) screen (Wang Y. et al., 2018). RXEG1 interacts with XEG1 by the extracellular LRR domain in the apoplast, and regulates XEG1-induced plant cell death and immune responses.

Using a genetic mapping approach, an *ELR* (elicitin response) gene has been identified from a population derived from the cross of *Solanum microdontum* genotype mcd360-1 (responds to INF1 with a cell death response) with *S. microdontum* ssp. *gigantophyllum* gig714-1 (does not respond to INF1) (Du et al., 2015). *ELR* encodes an LRR-RLP, and ELR mediates extracellular recognition of a broad range of elicitins exhibiting relatively low sequence similarity, suggesting that ELR recognizes elicitins most likely based on domain similarity but not a small conserved peptide. Moreover, cultivated potato transformed with the *ELR* gene exhibited enhanced resistance to *Phytophthora infestans*. Overall, the results suggested ELR as a potential cell surface receptor to mediate response to elicitins. However, the physical association of ELR with INF proteins remains unclear (Du et al., 2015). Interestingly, two intracellular proteins, a lectin-like receptor kinase (NbLRK) and a pepper calcium-binding protein (SRC2-1), have been shown to interact with *P. infestans* INF1 (Kanzaki et al., 2008) and *P. capsici* PcINF1 (Liu et al., 2015), and mediate *P. infestans* INF1 or PcINF1-induced cell death, respectively. These results correspond with the speculation that elicitins could be possibly transported into plant cells through clathrin-mediated endocytosis (Leborgne-Castel et al., 2008). Hence, it would appear that plants possess multiple mechanisms to recognize elicitins.

The three cell surface receptors RLP23, ELR and RXEG1 have extracellular LRRs but lack a cytoplasmic signaling domain. Two LRR receptor-like kinases (LRR-RLKs) SUPPRESSOR OF BIR1-1 (SOBIR1) and/or BRI1-ASSOCIATED KINASE-1/SOMATIC EMBRYOGENESIS RECEPTOR KINASE-3 (BAK1/SERK3) were shown to be essential for RLP23, ELR or RXEG1-induced cell death and immune responses that act as co-receptors to transduce signals to downstream elements. RLP23 forms a complex with SOBIR1 and recruits BAK1/SERK3 into a tripartite complex upon ligand binding (Albert et al., 2015); ELR associates with BAK1/SERK3 and mediates recognition of diverse elicitins from *Phytophthora* species (Du et al., 2015); RXEG1 associates with BAK1/SERK3 and SOBIR1 to transmit the XEG1-induced defense signal (Wang Y. et al., 2018).

BAK1/SERK3 and SOBIR1 appear to be general and central regulators of plant cell death and defense response induced by diverse apoplastic CDIPs besides nlp20, INF1 and XEG1. In *Arabidopsis*, *bak1* mutants showed a significantly reduced sensitivity to the CPP family CIDP BcSpl1, indicating that BAK1 plays an important role in the perception of BcSpl1 (Frías et al., 2011). In *N. benthamiana*, VIGS-mediated silencing of *BAK1* or *SOBIR1*, resulted in significant reductions of cell death induced by a number of apoplastic CDIPs, including BcXYG1 (Zhu et al., 2017a), BcXyl1 (Yang et al., 2018c), RcCD1 (Franco-Orozco et al., 2017), VdCUT11 (Gui et al., 2018), VdEG1, VdEG3 (Gui et al., 2017), VmE02 (Nie J. et al., 2019), MgNLP, Zt9, Zt11 or Zt12 (Kettles et al., 2017). These results indicate that BAK1/SERK3 and SOBIR1 are required for apoplastic CDIP-induced cell death.

Several cytosolic, or nucleo-cytoplasmic regulators have been shown to be important components in apoplastic CDIP-induced cell death and defense signal transduction pathways downstream of the cell surface receptor complexes. The ubiquitin ligase–associated protein SGT1 functions as a conserved component in both PTI and effector-triggered immunity (ETI) pathways. Silencing of *SGT1* in *N. benthamiana* suppressed cell death induced by *P. capsici* PcINF1 (Liu Z. Q. et al., 2016), INF1, INF2A (Huitema et al., 2005), PiNPP1.1, PsojNIP (Kanneganti et al., 2006), BcSpl1 (Frías et al., 2011), NIS1 (Yoshino et al., 2012), NcCDI1 (Franco-Orozco et al., 2017), or VmE02 (Nie J. et al., 2019). In addition, VIGS-mediated silencing analysis revealed that HSP70, HSP90 (Kanzaki et al., 2003; Kanneganti et al., 2006; Yoshino et al., 2012; Nie J. et al., 2019), COI1, MEK2, NPR1, TGA2.2 (Kanneganti et al., 2006), and Avr9/Cf-9-INDUCED F-BOX1 (ACIF1) (Li et al., 2019c) were required for cell death induced by certain apoplastic CDIPs.

## Concluding Remarks and Perspectives

An emerging number of studies have shown that many apoplastic CDIPs are required for pathogenicity, or contribute to the virulence of hemibiotrophic and necrotrophic pathogens, demonstrating the important role of these apoplastic CDIPs as essential virulence factors. On the contrary, apoplastic CDIPs were shown to elicit plant defense responses. Over the past decades, studies have documented that many apoplastic CDIPs are recognized by plants as PAMPs, being able to trigger *PR* gene expression, phytoalexin synthesis, MAPK phosphorylation, SA-, JA/ET-signaling pathways, as well as resistance against pathogens. The recent findings of extracellular recognition of NLPs, elicitins and XEG1 by plant RLPs, RLP23, ELR and RXEG1 (Albert et al., 2015; Du et al., 2015; Wang Y. et al., 2018), respectively, have greatly advanced our understanding of the roles of apoplastic CDIPs in plant-pathogen interactions. More importantly, the identification of receptors for apoplastic CDIPs provides valuable gene resources for engineering crops with broad and durable disease resistance (Albert et al., 2015; Du et al., 2015).

Currently, the majority of apoplastic CDIPs have been characterized based on demonstrations in dicot plants, especially based on transient expression in *N. benthamiana*, even many apoplastic CDIPs were identified from monocot pathogens. Particularly, recognition receptors and downstream components that have been identified remain restricted to dicot plants. Given the paramount importance of monocot cereal plants, such as rice, wheat and maize, as staple crops, it would be important to determine recognition of apoplastic CDIPs in monocot hosts, which could help engineer monocot cereal crops with broad-spectrum resistance against filamentous pathogens.

## Author Contributions

YL, YH, and SC wrote the manuscript. MQ, JC, XC, and XG contributed with comments and inputs. ZW and SC revised the manuscript with comments and inputs from all authors. All authors commented on the manuscript.

## Conflict of Interest

The authors declare that the research was conducted in the absence of any commercial or financial relationships that could be construed as a potential conflict of interest.
